# Prebiotics as a Tool for the Prevention and Treatment of Obesity and Diabetes: Classification and Ability to Modulate the Gut Microbiota

**DOI:** 10.3390/ijms23116097

**Published:** 2022-05-29

**Authors:** Ashwinipriyadarshini Megur, Eric Banan-Mwine Daliri, Daiva Baltriukienė, Aurelijus Burokas

**Affiliations:** Department of Biological Models, Institute of Biochemistry, Life Sciences Center, Vilnius University, Sauletekio Ave. 7, LT-10257 Vilnius, Lithuania; ashwinipriyadarshini.megur@bchi.stud.vu.lt (A.M.); eric.daliri@gmc.vu.lt (E.B.-M.D.); daiva.baltriukiene@bchi.vu.lt (D.B.)

**Keywords:** prebiotics, obesity, diabetes, gut microbiota, biotherapeutics, dietary fiber

## Abstract

Diabetes and obesity are metabolic diseases that have become alarming conditions in recent decades. Their rate of increase is becoming a growing concern worldwide. Recent studies have established that the composition and dysfunction of the gut microbiota are associated with the development of diabetes. For this reason, strategies such as the use of prebiotics to improve intestinal microbial structure and function have become popular. Consumption of prebiotics for modulating the gut microbiota results in the production of microbial metabolites such as short-chain fatty acids that play essential roles in reducing blood glucose levels, mitigating insulin resistance, reducing inflammation, and promoting the secretion of glucagon-like peptide 1 in the host, and this accounts for the observed remission of metabolic diseases. Prebiotics can be either naturally extracted from non-digestible carbohydrate materials or synthetically produced. In this review, we discussed current findings on how the gut microbiota and microbial metabolites may influence host metabolism to promote health. We provided evidence from various studies that show the ability of prebiotic consumption to alter gut microbial profile, improve gut microbial metabolism and functions, and improve host physiology to alleviate diabetes and obesity. We conclude among other things that the application of systems biology coupled with bioinformatics could be essential in ascertaining the exact mechanisms behind the prebiotic–gut microbe–host interactions required for diabetes and obesity improvement.

## 1. Introduction

The condition of obesity and diabetes has risen drastically in the last decade, leading to a public health emergency. In a recent study, 463 million people were estimated to suffer from diabetes worldwide and the number is expected to increase in the coming years [1]. Diabetes is typically preceded by insulin resistance, where insulin action in peripheral tissues including the liver, skeletal muscles, and adipose tissues are impaired. This results in reduced insulin-stimulated glucose disposal, reduced lipolysis rates, and decreased insulin-induced suppression of hepatic glucose production [2]. There is increasing evidence that disruption of the gut microbiota function and composition could contribute to the pathogenesis of metabolic diseases such as diabetes [3] and obesity [4,5,6]. Consequently, it is crucial to evaluate the cross talk between the gut microbial composition in the gut, the development of metabolic disorders, and the potential therapeutic strategies to prevent these metabolic syndromes.

The mammalian gastrointestinal tract (GIT) is home to trillions of microorganisms, collectively known as the gut microbiota (GM) [7]. The GM is defined as an ecological community of commensal microorganisms that live symbiotically and pathogenically in the gut [8]. Colonization of neonatal gut may start during birth [9]. GM represents a complex ecosystem, consisting of numerous diverse sets of microorganisms such as viruses, fungi, bacteria, archaea, and phages, deeply implicated in different functions of host metabolism [10]. The most abundant phyla consists of *Firmicutes* (64%), *Bacteroidetes* (23%), *Proteobacteria* (8%), and *Actinobacteria* (3%) [11]. GM makes a crucial contribution to the production of enzymes that are not encoded by the human genome, for example, the breakdown of polysaccharides, polyphenols, and the synthesis of vitamins [12]; is pivotal for human development and physiology [13]; and plays a vital role in regulatory functions in health and disease [14].

The composition of the GM differs between person-to-person and can fluctuate significantly within an individual [15]. Variation in GM composition could be caused not only by differences in the host’s genome, but also by environmental factors, such as antibiotic use, lifestyle, hygiene, and diet administration [16,17]. Significant alterations in gut microbial composition (dysbiosis) can be unfavorable and can predispose an individual to disease. For instance, acute and chronic disorders such as obesity, inflammatory bowel disease, irritable bowel syndrome, diabetes, colon cancer, and antibiotic-associated diarrhea have all been associated with dysbiosis [12,18,19].

Food is considered as a substrate that greatly contributes to the growth of GM and has a significant influence on its composition [20]. In 1980, it was proposed that definite components of the diet could promote the proliferation of specific bacterial strains inhabiting in the GIT, which are associated with the benefit of the host’s health [21]. The dietary intervention with prebiotics can be classified as dietary fibers; however, not all fibers can be considered as prebiotics [22]. Dietary modulation of GM with prebiotics has shown great potential as an agent to ameliorate and perpetuate a balanced microbial composition to improve health and well-being [23,24,25,26].

In this review, we discussed prebiotics, their classification, and the modulatory capacity of GM for health promotion in the host. We also discussed in vivo and in vitro studies and human clinical trials to provide better insight into the benefits of prebiotics on health. Finally, we focused on the therapeutic uses of prebiotics in the treatment/prevention of obesity and type 2 diabetes mellitus (T2DM).

## 2. Prebiotics

**Figure 1:**
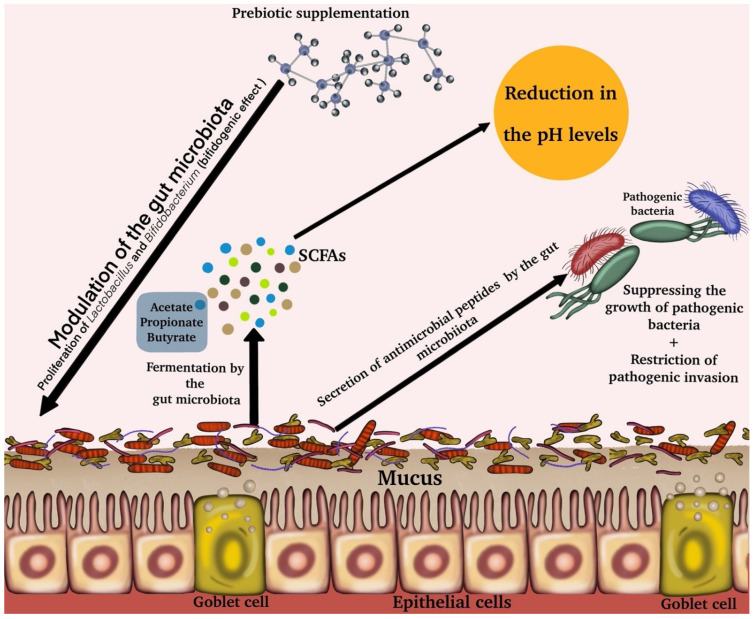
Mechanism of action of prebiotic supplementation. Prebiotic administration in a regular diet increases bacterial growth and functionality of specific species or genera, leading to modulation of the GM and showing a strong bifidogenic effect. The goblet cells play a key role in the production of mucus, which helps to protect the mucous membrane and form a layer in the colon that helps to reduce the inflammation caused by the bacterial interaction with intestinal epithelial cells. The modulated GM ferments prebiotics to form SCFAs (acetate, propionate, and butyrate), from which health benefits can be accrued. The production of antimicrobial agents and the reduction in the pH levels of the intestine due to prebiotic supplementation can suppress and restrict the growth of pathogenic bacteria, which can lead to positive health effects.

Prebiotics are a class of nutritional compounds categorized together, not necessarily by structural affinity, but by the potential to promote the growth and/or activity of specific beneficial bacteria (probiotics) in the GM. The concept of prebiotics came into recognition due to Glenn Gibson and Marcel Roberfroid in 1995 [23]. A prebiotic is known as “a non-digestible food constituent that beneficially influences the host by selectively promoting the growth and/or activity of one or a restricted number of bacteria in the colon, and thus improving the host health” [27]. In 2004, prebiotics were upgraded to include four criteria: (1) resistance to hydrolysis by mammalian enzymes, gastric acidity, and gastrointestinal absorption; (2) they should only be fermented by GM; (3) induce systemic or luminal effects that are beneficial to host health; and (4) selectively stimulate the growth and activity of GM associated with health and well-being [28]. The health benefits of prebiotics are diverse and include immune modulation through increased immune-regulatory interleukins and intestinal-specific immunoglobulins; reduction of pro-inflammatory interleukins [29,30]; and production of short-chain fatty acids (SCFAs) such as acetate, propionate, and butyrate [31] (Figure 1). SCFAs are carboxylic acids with aliphatic tails of one to six carbons that are produced by anaerobic fermentation of dietary fibers in the intestine by the GM [32]. SCFAs are an important indicator of bacterial fermentation in the colon and are known to improve the gut health by maintaining intestinal barrier integrity [33], mucus production [34], protection against inflammation, and reduction in colorectal cancer and obesity [35].

Among the abundant food ingredients available, some peptides and proteins, particular lipids, and non-digestible carbohydrates are components of prebiotics [36]. The chemical structures of these components are not absorbed in the upper part of the GIT or hydrolyzed by the digestive enzymes of humans. Hence, these ingredients are called colonic foods [37]. In colonic food, non-digestible carbohydrates are naturally occurring and meet all the criteria of prebiotics. These carbohydrates include non-starch polysaccharides, resistant starch, and non-digestible oligosaccharides [38]. However, not all of them are prebiotics [39]. In order to be classified as prebiotics, carbohydrates must fulfil the following criteria: (i) they are dietary fibers with a degree of polymerization (DP) between three and nine [40], and (ii) the endogenous enzymes produced in the small intestine should not hydrolyze them [41]. It should be taken into account that fermentation and fiber solubility are generally not curtailed [22].

Bacterial genera that promote health such as *Lactobacillus* and *Bifidobacterium* is proliferated by the administration of prebiotics, so that the fermented metabolites can be easily absorbed by the mammalian gut and have an influence on host physiology [42] (Figure 1). The prebiotics share several characteristics with dietary fiber, which includes partial or total resistance to digestion and fermentation by the GM. Due to its selectivity, prebiotics highlight the key condition to be demonstrated in an in-vivo experiment (including complex human or animal GM) using validated and relevant methodologies to quantify a wide variety of species that make up the GM [43].

Through characteristic and selective assimilation of prebiotics by subsequent fermentation, there is a production of SCFAs at high levels, having immunomodulation and metabolic effects on the host [44]. In this case, a reduction in the intestinal pH is also observed, creating an environment that competitively hinders the growth of pathogenic bacteria [45]. Some prebiotics prevent the adhesion of pathogenic microbiota to the GIT by mimicking an intestinal binding site [46] (Figure 1).

The application of prebiotics is well known in pharmaceuticals, and products for people with diabetes (as a natural sweetener) [47]. The large number of scientific data on prebiotics has focused on compounds associated with two major chemical groups: fructo-oligosaccharides and galacto-oligosaccharides [48]. They can be derived and/or extracted from food sources such as seeds, whole grains, legumes, chicory roots, Jerusalem artichokes, onions, garlic, and some vegetables [49], but in a recent study it was found that some aquatic plants (seaweeds and microalgae) contain prebiotics [50]. Prebiotics include a variety of forms such as fructo-oligosaccharides (FOS), galacto-oligosaccharides (GOS), human milk oligosaccharides (HMO), lactulose, lactosucrose, inulin, resistant starches (RS), arabinoxylans (AX), xylooligosaccharides (XOS), and pectin [24]. More attention has been given by researchers towards FOS as a prebiotic in improving human health [51].

## 3. Classification of Prebiotics

As mentioned above, there are many types of prebiotics that can be classified into different groups [52]. They differ in structure and can have a health benefit to the host through numerous different mechanisms [44]. Prebiotics also have the potential to modulate GM by selectively stimulating the growth of *Bifidobacteria* and *Lactobacilli*, by assimilation via beneficial GM and subsequent fermentation. In the fermentation process, these GM produce high levels of butyrate, isobutyrate, valerate, propionate, and acetate, which has various physiological functions in an organism [53]. The majority of prebiotics are mostly the subset of carbohydrate groups, more specifically, oligosaccharide carbohydrates. There are many relevant articles on oligosaccharide carbohydrates [54,55], but there are also few pieces of evidence showing that prebiotics are not only carbohydrates [56].

### 3.1. Inulin (Fructan)

Inulin-type prebiotics are members of an immense group called “fructans”. Fructans constitute a group of compounds that confine all naturally occurring plant oligosaccharides and polysaccharides in which one or more fructosyl–fructose linkages form the majority of glycosidic bonds [57]. Hence, they are the primarily polymers of fructose units. Fructans can also be characterized by the DP, which refers to the number of repeated units in a polymer or oligomer chain [58]. The category of fructans consists of inulin and oligofructose (FOS) [59] (Table 1).

Inulin is a collective term that comprises all linear fructans with β (2→1) fructosyl–fructose glycosidic bonds [60]; this specific type of glycosidic bond gives inulin its distinctive physiological and structural properties. Inulin-type fructans resist enzymatic hydrolysis by small intestine digestive enzymes and human salivary enzymes because of the beta configuration bonds between fructose monomers [61]. Chemically, the linear chain of inulin is either an α-D-glucopyranosyl-[β-D-fructofuranosyl](n-1)-β-D-fructofuranoside (GpyFn) or αβ-D-fructopyranosyl-[β-D-fructofuranosyl](n-1)-β-D-fructofuranoside (FpyFn) [62].

### 3.2. Fructo-Oligosaccharides (Fructan)

Another type of fructans i.e., FOS, is a natural component that can be found in plants [63]. FOS are commercially prepared from chicory in a hydrolysis reaction using inulinase and may also be derived in an enzymatic synthetic reaction via the transfer of fructosyl units from sucrose molecules [64]. When presented structurally, FOS consist of a sucrose molecule linked by a chain of 3–30 fructosyl units. FOS are oligomeric linear fructans with β-(2–1) or β-(2–6) fructosyl-fructose linkages with the first monomer of the chain either being α-D- glucopyranosyl or β- D -fructopyranosyl residue [60]. The DP of inulin is up to 60 and the DP of FOS is less than 10 [65] (Table 1).

### 3.3. Galactooligosaccharides

GOS are the product of lactose extension and are included among non-digestible oligosaccharides. They are arranged in two subgroups: (i) with excess galactose at C_3_, C_4_ and C_6_; and (ii) manufactured from lactose through enzymatic trans-glycosylation [66]. The mixture of the product depends on the reaction conditions and the enzymes used. β-galactosidase of various origins, such as *Aspergillus oryzae*, *Bacillus circulans*, and *Cryptococcus laurentii*, is used for the industrial production of GOS [67]. The general constituents of this oligosaccharide are from tri- to penta-saccharide with β (1→6), β (1→3), and β (1→4) linkages. This category of GOS is known as trans-galacto-oligosaccharide [68]. Culture studies of *Bifidobacteria* and most of *Lactobacilli* and enterobacteria, including some streptococci-metabolized trans-oligosaccharide, with *Bifidobacteria* showed robust growth [69]. There are some GOS derived from the isomers of lactose, due to influential factors such as the source of the enzyme, temperature, pH, and substrate concentration. They are also considered as prebiotics [70] (Table 1).

### 3.4. Human Milk Oligosaccharides

HMO are complex and non-digestible carbohydrates, recently classified as prebiotic substances. They are present in high abundance in maternal breast milk (10–15 g/L) [57,71,72]. The length of the HMO chain can vary from 3 to 15 carbohydrate units and is synthesized in the mammary gland [73]. The HMO concentration in the lactating mother is higher during the early stages and gradually decreases over time [74,75,76]. Structurally, HMO are composed of five monosaccharides: glucose, galactose, N-acetylglucosamine, fucose, and N-acetylneuraminic acid or sialic acid [77,78,79]. They are synthesized from a lactose core (galactose-β (1→4) glucose) by glycosyl transferases in the lactocyte. Some HMO are branched with a fucose or sialic acid monosaccharide residue attached to the lactose core via α1–2/3/4 and α2–3/6 linkages, due to the action of fucosyltransferases and sialyltransferases, respectively [80,81]. Among its several types, less than 50 HMO have a representative abundance in human breast milk. HMO 2′-fucosyllactose has been identified as the most abundant HMO in breast milk [82]. Breast milk due to its high levels of 2′-fucosyllactose has shown advantages for the infant because of its efficiency to promote an early high *Bifidobacteria*-dominated GM [83]. Several experiments conducted on the supplementation of HMO documented beneficial effects on the overall health of an individual, which includes modification of the GM [78,81,84], effects on immune development [78,85,86], anti-adhesive antimicrobial effects [87], and brain development [88,89] (Table 1).

### 3.5. Glucose-Derived Oligosaccharides

An example of glucose-derived oligosaccharide is polydextrose (PDX), which is non-digestible and widely used in the food industry [90]. PDX is a randomly bonded glucose polymer with an average DP of 12, but ranging from 2 to 120. This molecule contains the combination of α- and β-linked 1→2, 1→3, 1→4, and 1→6 glycosidic linkages [91]. PDX has been acknowledged as a soluble fiber that has beneficial effects on gut health, satiety, and postprandial glycemia [92]. Daily intake of 4–12 g of PDX has been found to have a large improvement in physiological functions without showing any adverse effect [93].

### 3.6. Resistant Starches

The starch that is resistant to the upper gut digestion is termed as RS [94]. RS cannot be digested by human pancreatic amylase in the small intestine, reaching the colon, promoting health benefits by producing a high level of butyrate, suggesting it to be classified as a prebiotic [39,95]. RS consumption has been related to improving the diabetes condition by reducing postprandial glycemic and insulinemic responses, and is also associated with decreased levels of cholesterol and triglycerides [96].

### 3.7. Pectic Oligosaccharides

Pectic oligosaccharides (POS) originate from a polysaccharide, known as pectin, which is a structural element of intracellular regions and cell walls of the plants and is vastly present in fruits and vegetable waste materials [97]. Chemically, POS are based on the extension of rhamnose or galacturonic acid, and different types of sugars (galactose, xylose, and arabinose) or ferulic acid are linked to the side chains [98]. In humans, gastric juice and saliva are not capable of degrading pectin. Furthermore, digestive enzymes like trypsin, pepsin, and rennet cannot breakdown pectin in the small intestine [99]. It has been reported that pectin undergoes slow fermentation and exhibits prebiotic effects by producing SCFAs [100]. It has been shown that pectic oligosaccharide has the potential to show bifidogenic effects [101]. Experiments conducted on pectic oligosaccharides revealed health benefits that include antiobesity, anticancer, and antioxidant properties [102].

**Table 1:** Summary of the structure and formula of prebiotics.

Abbreviation	Chemical Composition	DP	Chemical Formula	References
Inulin	linear chain of fructose with β(2→1) linkages	3–60	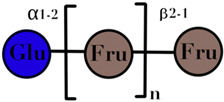 n = up to 100	[59,60]
FOS	linear chain of fructose with β(2→1) linkages	<10	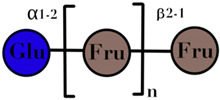 n = 1–5	[59,60]
GOS	Chain of galactosyl residues and a terminal glucose linked by β-(1–2), β-(1–3), β-(1–4), or β-(1–6) glycosidic bonds	2–8	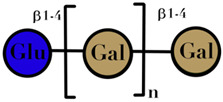 n = 1–4	[60,103]
HMO	composed of five monosaccharides: glucose, galactose, N-acetylglucosamine, fucose, and N-acetylneuraminic acid or sialic acid	<7	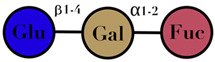 2′fucosyllactose 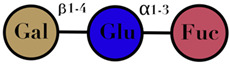 3′-fucosyllactose 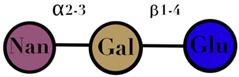 3′-sialyllactose 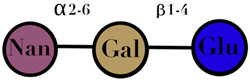 6′-sialyllactose 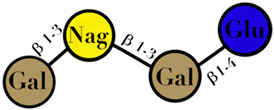 Lacto-N-tetraose 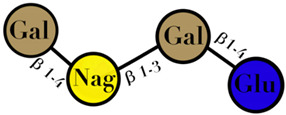 Lacto-N-neotetraose 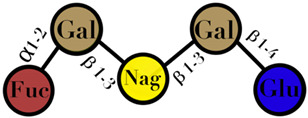 Lacto-N-fucopentaose I	[104,105]
Lactulose	consisting of galactose and fructose moieties	-	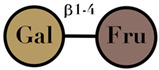	[106,107]
Lactosucrose	composed of galactose, fructose, and glucose monomers	-	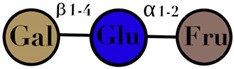	[108]
AX	β-1,4-linked D-xylopyranoside units substituted with arabinose residues on the c(o)-2 or c(o)-3 position	1–60	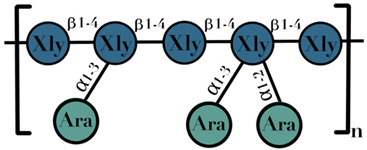	[109]
XOS	xylose moeities linked by β-(1→4) glyosidic bonds	2–4	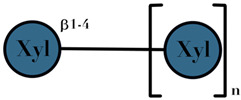 n = 2–4	[110,111,112]
Symbols used in Table 1: their meaning and chemical structure.		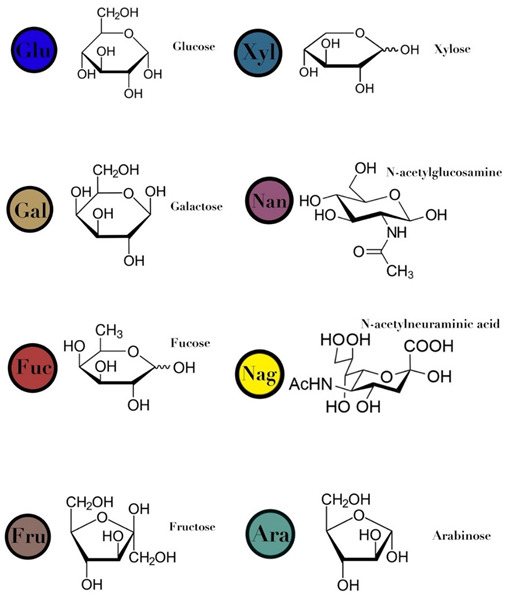		

### 3.8. Lactulose

Lactulose is a synthetically produced non-digestible ketose disaccharide that consists of galactose and fructose linked by a bond resistant to lactase [113]. Lactulose is extracted from lactose (milk sugar), chemically known as 4-O-β-d-galactopyranosyl-d-fructose, and the enzyme used for the biocatalytic production is β-galactosidase [114]. It is used medically for the treatment of constipation [115]. The human small intestinal mucosa does not have the enzymes to breakdown lactulose, and hence it reaches the large bowel unchanged [116]. Lactulose is metabolized by colonic bacteria to monosaccharides and then to methane, volatile fatty acids, and hydrogen [117]. In human studies, the lactulose have a significantly modified GM by increasing *Bifidobacterium*, *Lactobacillus,* and *Streptococcus,* and having favorable health benefits [81,118] (Table 1).

### 3.9. Lactosucrose

Lactosucrose is also known as galactosylsucrose, lactosylfructoside, and galactosucrose, and is synthetically produced trisaccharide, which is composed of galactose, fructose, and glucose monomers [119]. Raffinose, an isomer of lactulose, has a potential bifidogenic effect [120]. Lactosucrose is used as a commercial food supplement in many healthy foods and beverages with the intention of altering gastrointestinal functions and improving health [121]. Lactosucrose has shown promising effects as a bifidogenic compound modulating immune functions [122,123] (Table 1).

### 3.10. Arabinoxylans

Arabinoxylans (AX) are predominant non-cellulosic polysaccharides of cell walls in plants. AX were first identified by Hoffman and Gortner in 1927, as viscous gum in wheat flour [124]. Their structural properties, heterogeneity, and recovery depend on their location, which is strongly influenced by the other components of the cell wall [125]. AX are called as “pentosans” as they consist of pentoses xylose and arabinose. Chemically, it is heteroxylan consisting of a backbone of β-1,4-linked D-xylopyranoside units substituted with arabinose residues on the c(o)-2 or c(o)-3 position [126]. The DP of AX is between 1 and 60 [109]. AX have the potential to show high technological importance. There are several biological studies that have been reported on the behalf of AX, including antioxidant activity [127], cholesterol-lowering agents [128], blood sugar modifiers [129], and immunity enhancers [130] (Table 1).

### 3.11. Xylooligosaccharides

Xylooligosaccharides (XOS) or xylan are to be considered as the second most abundant biopolymer in the plant kingdom. These are the sugar oligomers of β-1,4-linked xylose (a pentose sugar) found naturally in food sources such as honey, bamboo shoots, fruits, vegetables, and milk [131]. On the basis of substituted groups, xylan can be categorized into three classes: (i) glucuronoxylan, (ii) neutral arabinoxylan, and (iii) glucuronoarabinoxylan [132].The DP of the XOS used in commercial food products ranges from 2 to 10 [133]. The complete utilization of XOS is based on the activities of a number of enzymes, including β-xylosidase, α-glucuronides, and acetyl esterases released by different strains of GM, and produces SCFAs [134]. XOS have shown a bifidogenic effect [135], with the support of in-vivo animal studies, and offers modification in the composition and activity of the GM [136] (Table 1).

Excess consumption of prebiotics can promote severe discomfort in an individual, therefore, optimal intake is necessary [137] (Table 2).

## 4. Efficacy of Prebiotics on Gut Microbiota Composition: In Vivo and In Vitro Studies

The experiments conducted on the administration of prebiotics have shown selective changes in the GM composition. Different categories of prebiotics can stimulate the growth of various indigenous bacterial communities in the GM. Collective evidence from animal model trials, human studies, and in-vitro modeling systems has concluded that they affect the composition of GM, leading to proliferation in health-promoting organisms such as *Bifidobacteria* and *Lactobacilli* [146,147,148]. Prebiotics have ameliorative properties such as maintaining intestinal integrity and homeostasis, production of SCFAs, and regulation of gastrointestinal transit [115]. Indeed, it has been suggested that the use of prebiotics should have ameliorative properties on gastrointestinal diseases like irritable bowel disease, Chron’s disease, and ulcerative colitis [149]. Selective stimulation of GM growth and/or activity is potentially associated with health protection and well-being [24,150].

### 4.1. Inulin

Inulin is a non-digestible oligosaccharide that is fermented by the GM and has resistance to the degradation by the human digestive enzymes. It reaches the colon almost as an intact molecule and acts as a fermentable substrate for GM [62]. In vivo and in vitro studies on inulin concluded that it has selective stimulation of bacterial growth; this has been observed in numerous studies carried out either in defined pure culture fermentation or by using human feces [151,152,153]. Inulin supplementation for 19 days to a group of 10 elderly women with a dose beginning at 20 g/day from days 1 to 8 and gradually increasing to 40 g/day during days 9 to 19, showed a significant increase in *Bifidobacteria* that can be utilized during fermentation, and a decrease in the number of *Enterococci* and *Enterobacteriaceae*, while no statistically significant changes were observed in *Bacteroides*, *Clostridia*, or *Faecalibacterium prausnitzii* [154]. In another study conducted on 10 healthy volunteers with inulin supplementation of 8 g per day for 14 days, a significant increase in *Bifidobacteria* was shown. In this case, a number of *Clostridia* increased also, but the magnitude of *Clostridia* was one tenth of *Bifidobacteria*. These data supported a bifidogenic effect of inulin [155]. Importantly, inulin fermentation leads to the production of SCFAs. In an experiment conducted on rats cecum (colonic part of the GIT), it was demonstrated that inulin has significantly higher efficiency in producing SCFAs compared with other dietary fibers [156] (Table 3).

### 4.2. FOS

FOS have great potential as ingredients due to their prebiotic activity and low caloric value. Gibson and Roberfroid [23] showed the bifidogenic characteristics of FOS using 15 g per day as dietary supplementation. The GM was modulated and there was a significant decrease in the number of *Bacteroides*, *Fusobacterium,* and *Clostridium*. Therefore, it was concluded that FOS is better utilized by *Bifidobacteria*, and, on the other hand, they can cause unfavorable changes for harmful bacteria in the GIT [23].

It was verified that the addition of NeosugarR (a trade name for fructooligosaccharide) to the human diet, i.e., 15 g per day, can cause a 10-fold increase in the population of *Bifidobacteria* in the large intestine [138]. In addition to its bifidogenic property, the regular and adequate intake of FOS has beneficial effects in the case of disorders associated with obesity, diarrhea, osteoporosis, atherosclerotic, gastrointestinal disorders, cardiovascular, and T2DM diseases [157]. The fermentation of FOS by GM generates SCFAs and organic acids that decrease luminal pH, thereby enhancing the bioavailability of nutritionally important minerals [158]. It was also found that a diet supplemented with FOS promotes the production of butyrate, which influences lipid metabolism in humans [159] (Table 3).

### 4.3. GOS

GOS are a type of non-digestible fiber with prebiotic activity [133], which has also been demonstrated by a dynamic in-vitro colon model and the ^13^C labeling technique with GOS consumption. The results showed an increase in *Bifidobacterium longum*, *B. bifidum*, *B. catenulatum*, *Lactobacillus gasseri*, and *L. salivarius*, but changes in numbers of *Enterobacteriaceae* (a family of Gram-negative bacteria that includes some harmless symbionts) and several familiar pathogens, such as *Salmonella*, *Yersinia pestis*, *Klebsiella*, *Escherichia coli*, and *Shigella*, were rather negligible [160]. In another study, the prebiotic activity of GOS was analyzed by pyrosequencing of fecal samples from healthy human volunteers with GOS administration. The data obtained showed a statistically significant increase in *Bifidobacteria* and *Faecalibacterium prausnitzii*, and a decrease in *Bacteroides* [161]. It was also concluded that 90% of GOS resist digestion in the upper GIT and then enter the colon, which then get intact to the tract and act as fermentation substrates for the resident microbiota [162] (Table 3). An in-vitro study showed that the fermentation of GOS by GM generates SCFAs and organic acids that decrease luminal pH, thereby enhancing the bioavailability of nutritionally important minerals [163]. Interestingly, GOS administration showed anxiolytic effects in both animals [164] and humans [165] (Table 3).

### 4.4. HMO

One of the multifarious functions of HMO is that they act as prebiotics and stimulate the colonization of beneficial GM [166]. In vitro studies provided strong evidence that HMO promotes the growth of selective *Bifidobacteria* [78]. *Bifidobacterium longum subsp. infantis* proliferates well on 2′-FL, as the sole source of carbohydrates [81,84,167,168]. These *Bifidobacterium longum subsp. infantis* produce SCFAs, which create an environment that favors the growth of commensal bacteria and prevents the adhesion of pathogenic bacteria [169]. Some structures of HMO are similar to the intestinal epithelial cell surface glycan receptors, which serve as decoy receptors to prevent pathogen binding and increase pathogen removal [78]. A study on HMO supplementation suggested that breast-fed infants have a higher number of *Bifidobacteria* compared to the formula-fed infants [170]. In a human study, investigation into the interaction between *Bifidobacteria* and *Eubacterium hallii* demonstrated that *E. hallii* consume acetate, lactate, and 1,2-propanediol (which are the products formed by the fermentation of HMOs by *Bifidobacteria*) and eventually lead to the production of butyrate and propionate [171] (Table 3). On the other hand, the study conducted on bioengineered 2′-FL showed inhibition of the adhesion of *Campylobacter jejuni*, *Salmonella enterica*, *E.coli,* and *Pseudomonas aeruginosa* to an intestinal human cell line [172]. Research on HMO, specifically 2′-FL, has shown that it is even more potent than standard commercial prebiotics, such as FOS, and has many different functions, including immune, GM, and cognition benefits [173] (Table 3).

### 4.5. PDX

In vitro studies have indicated that PDX has all the characteristics to be a prebiotic [174,175]. It has been shown that daily intake can beneficially modify the composition and activity of GM. In a study in humans, PDX favored intestinal function and improved the ease of bowel movement. Furthermore, it inhibited the absorption of glucose in the small intestine and the fermentation for the production of SCFAs in the large intestine favoring the reduction of gut pH [176]. Supplementation with PDX in healthy humans with a dose of 8 g per day for 3 weeks showed a significant increase in the number of *Ruminococcus intestinalis*, the main producer of butyrate, and slow fermentation of PDX in the colon was observed [177]. Another study carried out in healthy adult males with 21 g of PDX supplementation per day significantly suppressed the number of phylum *Firmicutes* and significantly increased the number of bacteroidetes when compared to the control group [174]. These data concluded that PDX supplementation had a positive impact on the bacterial composition of GM (Table 3).

### 4.6. RS

A number of studies demonstrated that RS is capable of modifying the GM composition towards the heath benefit of the host. An experiment carried out in mice for 8 weeks showed that mice fed with diets containing high amylose RS2 (one of the types of RS) were colonized by higher levels of *Bifidobacterium*, *Akkermansia,* and *Allobactum* [178]. The nutritional intervention study revealed that RS, when supplemented in the diet, can induce a 10-fold increase of gut *Bifidobacteria* [179]. On the other hand, one of the byproducts of RS is SCFAs. A study carried out on rats for 12 weeks treated with two concentrations of RS (0 and 27% weight of diet) showed an increase in propionate, butyrate, and acetate [180] (Table 3).

### 4.7. POS

POS is a new class of prebiotics that derives SCFAs from the GM fermentation [181]. In an in vitro study, the POS from the citrus peel and sugar beet pulp were fermented by the human fecal samples, leading to an increase in the bacterial population of eight different groups. POS from sugar beet showed the highest bifidogenic effect and utmost SCFAs concentration. On the other hand, the POS from citrus peel showed an increase in the population of *Lactobacillus* [182]. In a recent study, it was concluded that the concentration of SCFAs was higher in the POS supplementation, when compared to FOS [183] (Table 3).

### 4.8. Lactulose

An investigation conducted on lactulose degradation determined that human and calf β-galactosidases do not degrade it [184]. An in-vitro study performed using fecal samples on agars, and an analysis of enzymes produced and putrefactive compounds of lactulose fermentation, concluded a selective and significant increase in *Bifidobacteria*, decreasing the abundance of streptococci, bacteroides, *C. perfringens,* and *Lactobacilli* [185]. Studies carried out on humans demonstrated that lactulose selectively and significantly modifies GM by increasing *Lactobacillus*, *Bifidobacterium,* and *Streptococcus* [186] (Table 3).

### 4.9. Lactosucrose

Strong evidence has been observed in the administration of lactosucrose, selectively promoting the number of *Bifidobacteria* in in-vitro and in-vivo studies on animals and humans [187,188,189]. Lactosucrose fermentation was evaluated using *Bifidobacterium*, *Lactobacillus*, and *Streptococcus* probiotic strains in the in-vitro study, and the results led to the growth of four bacterial strains: *Lactobacillus casei, Lactobacillus reuteri, Lactobacillus acidophilus,* and *Streptococcus salivarius* [187]. Animal studies have shown a significant increase in *Lactobacilli* and *Bifidobacteria*, while restraining the levels of pathogens, such as *Clostridium perfringens*, *Staphylococci,* and *Bacteroidaceae*, after the consumption of lactosucrose [122,185]. Lactosucrose fermentation by the GM produces SCFAs and shows a consequent reduction in the pH of fecal contents [190,191]. An in-vitro study on different fish species such as *Pagrus major*, *Cyprinus carpio* L., and *Oncorhynchus mykiss* showed that the lactosucrose fermentation results in the production of SCFAs and gases, concluding that the lactosucrose can also be fermented in herbivorous, omnivorous, and carnivorous fishes [192,193,194] (Table 3).

### 4.10. AX

AX are not digested by the enzymes produced by the GIT, thus these provide the carbon source for the GM that inhibits the large bowel [195]. Many experiments have been performed on the regular supplementation of AX, resulting in an enhancement in the proliferation of the growth of health-promoting bacteria. In-vitro studies of AX, implemented in anaerobic batch cultures inoculated with human feces, demonstrated that fermentation of wheat endosperm AX resulted in the production of acetate, propionate, and butyrate [196]. In the in-vitro digestibility test carried out on pigs, it was established that only 15% of the ingested AX is recovered in the feces, while the major fraction of AX is fermented in the cecum, which represents the high fermentability of AX [197] (Table 3).

**Table 3:** Prebiotic efficiency in modulating the GM.

Prebiotics	Model	Strategy/Duration of Feeding	Dose Supplemented	Form	No. of Applications	Re-Calculated Dose *	Fecal Microbial Changes Relative to Control	Reference
Inulin	17 elderly women (mean age = 76.4 years body weight not reported)	8 days, (3 days adaptation) Feeding was continued for 8 days	20 g/day and increased to 40 g/day	Dissolved in drinking water	Once/day	285.7 mg/kg/day and increased to 571.4 mg/kg/day	significant ↑ in *Bifidobacteria* ↓ in Enterococci and *Enterobacteriaceae*	[154]
	10 healthy volunteers (age = between 20 and 55 years Body weight not reported)	14 days	8 g/day	Dissolved in drinking water	Twice/day	114.3 mg/kg/day	significant ↑ in *Bifidobacteria* ↑ in the number of Clostridia	[155]
	Germ-free adult male Fischer rats (age = 10 weeks and body weight = 280 g)	8 weeks	1.84 g/day of the diet	Mixed with chow	During the day	6.57 g/kg/day	significant ↑ in producing SCFAs	[156]
FOS	Male Wistar rats (age = 2 months and body weight 403.2 ± 48.1 g)	7 days	8% of the diet	Mixed with chow	During the day	3.4 g/kg/day	↑ the bioavailability of nutritionally important minerals	[158]
FOS + GOS	10 Male C57BL/6J mice (age 8 weeks old mice; mean body weight = 28 g)	10 weeks	0.3 –0.4 g/mouse/day	Dissolved in drinking water	During the day	1.1–1.43 g/kg/day	↑ *Akkermansia* abundance	[164]
GOS	18 healthy human (age and body weight not indicated)	3 weeks	2.5 g/day 5 g/day, 10 g/day	Administered in edible chews	Once/day	35.7 mg/kg/day 71.4 mg/kg/day, 142 mg/kg/day	significant ↑ in abundance of *Bifidobacteria* and *Faecalibacterium prausnitzii*, ↓ in *Bacteroides*	[161]
	Mud crab (age not reported and body weight 63.6 ± 8.8 g)	24 h	0.05 g/day	Dissolved in water	During the day	786 mg/kg/day	↑ *Bacteroidetes*	[163]
PDX/FOS	77 Children (age 5.8 ± 1.3; body weight not reported)	2 weeks	4.17 g PDX + 0.45 g FOS	Dissolved in drinking water	Once/day	PDX 200 mg/kg/day + FOS 22 mg/kg/day	↑ in number of *Bifidobacterium* and *Lactobacillus*	[176]
PDX	20 Healthy men (Age = 27.5 6 ± 4.33; body weight = 86.26 ± 13.48 kg)	21 days	21 g/day	Mixed in bar	Once/day	243.4 mg/kg/day	↑ in number of *Faecalibacterium, Phascolarctobacterium,* and *Dialister*	[175]
	15 Healthy volunteers (age = 18–50 body weight not reported)	3 weeks	8 g/day	Powder	Once/day	243.5 mg/kg/day	↑ *Ruminococcus intestinalis*, *Clostridium clusters I, II* and *IV*, significantly ↓ levels of *Lactobacillus* and *Enterococcus* group	[177]
RS	6 Male C57BL/6J mice (18–20 month old and body weight not reported)	8 weeks	0.54 g/day	Mixed with chow	During the day	18 g/kg/day	↑ in number of *Bacteroidetes, Bifidobacterium* and *Akkermansia species*	[178]
	Sprague-Dawley rats (age 6 weeks and body weight not reported)	12 weeks	27% of the diet	Mixed with chow	During the day	18 g/kg/day	↑ in SCFAs	[180]
POS	Pigs’ fecal inoculum (age 4 years and the mean body weight 233.0 ± 10.02 kg)	48 h	9 g/of POS to 1 mL of inoculum	Mixed with the chow	-	-	↑ in SCFAs	[183]
Lactulose	12 healthy volunteers (age = (24 to 31 years and body weight not reported)	4 weeks	20 g/day	Mixed with chow	Twice/day	285.7 mg/kg/day	↑ in number of *Bifidobacterium* and *Lactobacillus.*	[186]
Lactosucrose	Red seabream *Pagrus major* (age and body weight not reported)	9 months	20 mg/kg/day	Mixed with chow	Once/day	20 mg/kg/day	↑ production of SCFAs	[192]
	8 Shepherd dogs (body weight = 22 to 32 kg; mean age = 13.5 months)	2 weeks	1.5 g/day	Mixed with chow	Twice/day	55.6 mg/kg/day	↓ in the levels of f *Clostridium pefringms* ↑ *Bifidobacterium*	[122]
	16 Broiler chickens (20–62 days and body weight not reported)	62 days	825 mg/day	Mixed with chow	During the day	458 mg/kg/day	↑ in the number of *Bifidobacterium* ↓ the number of *Bacteriodaceae*; *Staphylococci;* and total anaerobic bacteria, *C. perfringens*	[198]
	8 Cats (Mean agae + 7; body weight 3.5 kg)	2 weeks	50 mg of lactosucrose/day	Mixed with the chow	During the day	14 mg/kg/day	↑ in *Lactobacilli* and *Bifidobacterium* ↓ in *Clostridium perfringens*, clostridia, *Spirochaetaceae,* and *Enterobacteriaceae*	[185]
AX	10 human children (mean age, 3 years, 7 months body weight not reported) (in vitro)	48 h	10 g/liter	Dissolved in drinking water	-	--	↑ in number of *Lactobacillus*	[196]
XOS	12 healthy adult women (mean age for women = 33.6 years and body weight not reported) and 11 healthy men (mean age = 30.1 and body weight not reported)	8 weeks	1.4 g/day or 2.8 g/day	Capsule	Once/day	20 or 40 mg/kg/day	↑ *Bacteroides fragilis*, ↑ *Bifidobacterium*	[199]
	13 elderly human (body weight = 58.6 ± 10.1 kg body weight not reported)	3 weeks	4 g/day	Mixed with chow	Once/day	68.3 mg/kg/day	↑ in number of *Bifidobacterium* species	[200]

↑—Increase, ↓—Decrease, * unless indicated, the average adult human weight was estimated as 70 kg and the average rat weight was estimated to be 280 g.

### 4.11. XOS

Animal studies have furnished evidence that oral administration of XOS remarkably increases fecal weight, bone properties, fecal moisture, and number of *Bifidobacteria*, with a parallel increase in SCFAs production in mice [201], rats [202], and humans (elderly) [200]. A recent study on a healthy human adult demonstrated that XOS intake increases *Bifidobacterium* counts without affecting the number of *Lactobacillus* [199]. The potential of *Bifidobacteria* to metabolize XOS is based on the activity of their xylan-degrading enzyme systems. Human study on the prebiotic XOS and their effects on modulating the GM in vivo is limited, particularly regarding the efficiency (Table 3).

Prebiotics are also able to remodulate the composition of the GM. Compared to a different category of prebiotics, only the fructans (inulin and FOS), GOS, and lactulose had highly selective effects on human GM modification [203]. As mentioned before, fermentation products of prebiotics such as SCFAs also have modulatory effects on the gut pH [204]. The pH alteration can have an influence on the population of acid-sensitive species, such as *Bacteroides*, and promote butyrate formation by *Firmicutes* [205].

## 5. Prebiotics for the Treatment of Obesity and Diabetes

Globally, the population of diabetes patient is increasing, imposing a great social and economic burden on public health [206,207]. T2DM is a chronic metabolic syndrome of abnormal lipid and glucose metabolism that leads to neuropathy, retinopathy, leg ulcers, and gangrene [208]. The factors that could have an impact on T2DM development are obesity, genetics, smoking, age, hypertension, and sedentary lifestyle [207]. In recent studies, it has been proposed that the remolding of the GM composition from obesity could lead to the pathogenesis of T2DM [209,210,211,212,213].

**Figure 2:**
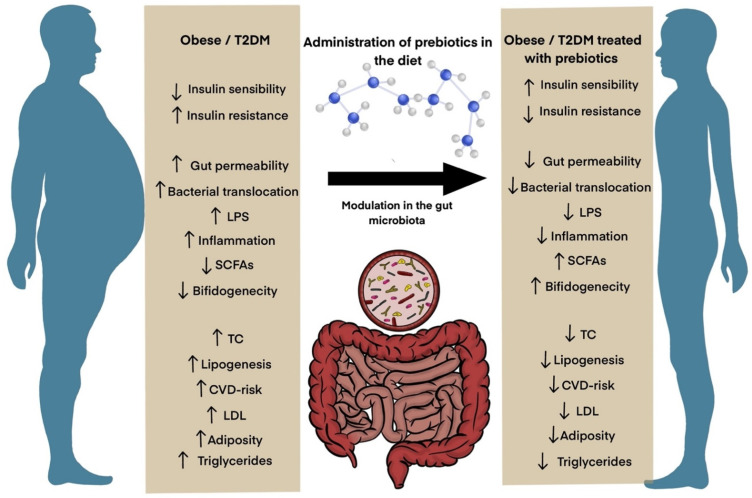
An overview of the improvement in the health of obese/T2DM patients treated by modulating their GM using prebiotics supplementation in a regular diet. Administration of prebiotics has the potential to modulate GM composition in patients suffering from T2DM and obesity and can be used as a therapeutic approach to cure the adverse effects of metabolic diseases. The daily intake of prebiotics in a designed diet has a major influence on GM by decreasing gut permeability, bacterial translocation, and reducing LPS-induced inflammation. However, this diet increases SCFAs and bifidogenecity in the gut, leading to lower TC levels, lipogenesis, LDL triglycerides, and adiposity, eventually resulting in lower risk of cardiovascular diseases.

As mentioned above, the two dominant bacteria groups in human GIT are *Bacteroidetes* and *Firmicutes* [209]. A link between obesity and GM composition has been reported in humans, showing an increase in the number of *Firmicutes* and a decrease in the diversity of *Bacteroidetes* [213] (Figure 2).

Prebiotics have gained a considerable place in the management of obesity and diabetes due to their ability to modulate GM composition, thereby affecting the status of GIT and exerting anti-diabetic effects [214,215]. As prebiotics consist of different forms, their supplementation can be considered as a dietary therapy for the prevention and treatment of T2DM [216], and also in the fight against obesity by affecting food intake and appetite and metabolic activities [10] (Figure 2).

FOS have numerous desirable characteristics such as low calories, safety for diabetics, no carcinogenicity, and bifidus-stimulating functionality [65]. Due to these properties, FOS are considered a functional food ingredient that improves health status [217]. Increasing studies demonstrated the functional properties of FOS including the reduction of blood glucose levels, cholesterol levels, and lowering of blood pressure [218,219,220] (Table 4).

Meanwhile, inulin as a prebiotic has shown mixed results on the glycemic scale [221,222]. A study carried out on 54 middle-aged (between 35 and 65 years) healthy adults (men and women) as a double-blind, randomized, placebo-controlled parallel groups with 10 g of inulin supplementation for 8 weeks did not show any significant changes in the body weight [223]. A decrease in plasma insulin level was observed after 4 weeks of treatment and remained lower up to the 8^th^ week, along with lower plasma triglyceride concentrations. Total cholesterol (TC) was lower in the inulin-supplemented group when compared to the placebo group. The study concluded that inulin supplementation may influence the degradation of triglyceride-rich lipoprotein particles [223]. In human trials conducted on obese women treated with inulin, greater proportions of *Bifidobacterium* and *Faecalibacterium* were observed, an effect that coincided with reduced fat mass and serum lipopolysaccharide [224]. The important role of *Bifidobacterium* in the fight against obesity has recently been demonstrated by *Bifidobacterium longum* both in preclinical obesity models and in humans [225].

**Table 4:** Effect of different prebiotics on the treatment of obesity and diabetes in animal and human studies.

Prebiotic Used	Tested Species	Dose	Re-Calculated Dose	Period	Outcomes	Reference
FOS	27 women with Type-2 diabetes, age = 20–65 years; 76.0 (12.2)	10 g/day	131.6 mg/kg/day	8 weeks	↓ Fasting plasma glucose (19.2 mg/dL; 9.50%), glycosylated hemoglobin (1.0%; 8.40%), interleukin-6 (1.3 pg/mL; 8.15%), tumor necrosis factor-α (3.0 pg/mL; 19.80%) and plasma lipopolysaccharide (6.0 EU/mL; 21.95%).	[220]
FOS	10-week-old C57BL/6J mice, body weight not reported	0.3 g/mouse/day		8 weeks	plasma TG, LPS ↑ plasma glucagon-like peptide-1 and colon proglucagon mRNA ↑ colon L-cells number	[226]
GOS	6 rats alloxan-induced diabetic rats, 6 weeks old; Average weight = 90 g	100 g/kg of diet	1.11 g/kg of diet	42 days	↑ level of antioxidative enzymes ↓ blood glucose, lipid profile, serum urea ↓ fecal coliform count	[227]
	Human (women with overweight age 18–65 years and body weight not reported)	5.5 g/day of GOS	5.5 g/kg/day	12 weeks	↓ fasting insulin levels, triglycerides, TC, and HDL cholesterol ↓ in fecal calprotectin	[228]
PDX	Rats (Wistar rats age not reported and body weight 43.0 ± 4.5 g)	5 g/100 g diet	5 g/100 g diet	60 days	↓ the of triglyceride (17%) lowered the hepatic cholesterol showed lower serum malondialdehyde	[229]
RS	Human (over weight and obese adults—11 men and 22 women age 18–69 years and body weight not reported)	15 g/kg/day of HAM-RS2 v. 30 g/kg/day HAM-RS21	15 g/kg/day of HAM-RS2 v. 30 g/kg/day HAM-RS21	4 weeks	↑ insulin sensitivity by insulin-modified intravenous Glucose Tolerance Test	[230]
	Human (diabetic adults age = 55 ± 2.4 years body weight not reported)	40 g/day of	571.4 mg/kg/day	12 weeks	↓ postprandial glucose by meal tolerance test ↑ glucagon-like peptide-1 ↓ tumor necrosis factor α	[231]
Lactulose	Human (patients with obesity age and body weight not reported)	8.2 g/day	-	2 days	↓ mean daytime glucose and insulin	[232]
AX	Rats (wild type rats with high cholesterol diet age 7 weeks body weight not reported)	8% corn arabinoxylan	5.8 g/kg/day	20 days	↓ uptake of cholesterol from the diet ↓ serum cholesterol levels abbreviated cholesterol accumulation in the liver	[233]
	Human (T2DM); mean age = 55 years and body weight not reported)	49.2 g/day	702.9 mg/kg/day	35 days	↓ fasting serum glucose levels. ↓ serum glucose and insulin level 2 h after oral glucose intake	[234]
XOS	Rats (Male Wistar rats treated with streptozotocin to induce diabetes, age = 8 weeks; body weight = 180 ± 8 g)	0.325 g/day	1.81 mg/kg/day	5 weeks	↓ diabetic weight loss ↓ serum glucose, triglycerides	[235]

↑—Increase, ↓—Decrease.

XOS studies indicated that they have the potential to reduce serum cholesterol and triglycerides levels, which are the main risk factors for diabetes. The administration of XOS to wild-type rats for 28 days showed a reduction in LDL levels, TC in serum, triglycerides, and body weight [236] (Table 4).

The consumption of RS improved insulin sensitivity in subjects with metabolic syndrome and appears to have a favorable effect on insulin sensitivity [231] (Table 4).

In summary, prebiotics show efficiency not only in modulating or restructuring and stabilizing the host microbiome, but also in the regulation of many mechanisms associated with the development and metabolic consequences of obesity. Furthermore, prebiotics should be enriched in popular foods, increasing the chances of consistent consumption and improving overall health. At least, dietary prebiotic supplementation represents a safe, well-tolerated, and inexpensive therapeutic approach and should be considered as a potential therapy for the treatment and prevention of T2DM and obesity (Figure 2).

## 6. Conclusions and Future Perspectives

Recent studies have proven that increased inflammatory state (as seen in obesity and diabetes) has a paramount influence on glucose metabolism, and eubiosis ensures appropriate immune responses. This implies that the implementation of appropriate GM modulatory strategies could be a new and promising therapy against metabolic diseases. Meanwhile, the appropriate dosage, duration of treatment, and long-term effects of the intervention of different prebiotics remain unknown. For this reason, more clinical trials are needed before prebiotics can be rationally suggested for the prevention and/or treatment of obesity and diabetes.

Although in-vivo and in-vitro studies conducted on animals and humans revealed that many prebiotics increase the growth of *Bifidobacterium* spp., and *Lactobacillus* spp. and cause a diverse change in the *Bacteroidetes* and *Firmicutes* phyla, it is still not fully understood how these carbohydrates interact with the GM with their widely diversified structures. Further research is required to reveal the mechanisms of these carbohydrates’ structures on the GM and the host. In addition, it is well established that GM ferments the prebiotics, leading to the formation of SCFAs and acidification of the colonic contents. These by-products formed by the fermentation process play an extensive role in maintaining the host’s health and ameliorate diseases. Despite advances in our understanding of prebiotics, there remain numerous knowledge gaps concerning the SCFAs molecular signaling mechanisms and their association with prebiotic chemical composition and structural conformations, along with their modulatory effects at the genetic, cellular, organelle, and systemic levels.

Meanwhile, the application of systems biology coupled with bioinformatics could be a powerful strategy to unveil mechanistic insights into the action of prebiotics on the gut microorganisms and lead to an understanding of how these compounds (and their metabolites) alter both microbial and host metabolic functions at the molecular level. These insights and population-based studies could uncover new strategies to improve dietary relevance and clinical efficacy.

## Figures and Tables

**Table 2:** Recommended intake of prebiotics.

Prebiotic	Doses Suggested	Reference
Inulin	2–12 g/day	[138]
FOS	12.5–20 g/day	[139]
GOS	2–20 g/day	[140]
HMO	10–20 g/day	[141]
PDX	4–12 g/day	[93]
RS	10–15 g/day	[142]
POS	10–20 g/day	[143]
Lactulose	10–30 g/day	[144]
Lactosucrose	Not estimated	-
AX	Not estimated	-
XOS	1–5 g/day	[145]

## Data Availability

Not applicable.

## Abbreviations

GIT—gastrointestinal tract, GM—gut microbiota, SCFAs—short-chain fatty acids, DP—degree of polymerization, FOS—fructo-oligosaccharides, GOS—galacto-oligosaccharides, HMO—human milk oligosaccharide, PDX—polydextrose, RS—resistant starch, POS—pectin oligosaccharides, AX—arabinoxylans, XOS—Xylooligosaccharide, T2DM—Type-2 diabetes mellitus, TC—total cholesterol, LDL—low-density lipoprotein, HDL—high-density lipoprotein.
